# Approximate symmetry in the third reported structure of a metal complex of l-DOPA

**DOI:** 10.1107/S2053229621007452

**Published:** 2021-07-30

**Authors:** Carolyn Pratt Brock

**Affiliations:** a Department of Chemistry, University of Kentucky, 505 Rose Street, Lexington, KY 40506-0055, USA

**Keywords:** commentary, L-DOPA, approximate symmetry, MATCH

## Abstract

Although l-DOPA is a major drug that has been used for more than 50 years to treat the motor symptoms of Parkinson’s disease, the Cambridge Structural Database contains only two structures in which it is coordinated to a metal. Why are there not more? The new structure is also notable for an approximate 4_2_ axis.

The 2021 article ‘*cis*-Bis(l-DOPA-κ^2^ *N*,*O*)copper(II) monohydrate: synthesis, crystal structure, and approaches to the analysis of pseudosymmetry’ by O’Brien *et al.* (2021 ▸) is notable for two reasons. First, it reports only the third crystal structure of a metal complex containing the medicinally important ligand l-DOPA (3,4-dihy­droxy-l-phenyl­alanine). Second, the article includes a tutorial on finding approximate symmetry relationships between mol­ecules that are chemically equivalent but crystallographically independent. Identification of approximate symmetry in mol­ecular crystals is not yet a solved problem; rather, it is an area of considerable recent activity. Rekis (2020 ▸) proposed a method for finding approximate inversion centers; Brock & Taylor (2020 ▸) described software for finding approximate translations; Brock (2020 ▸) identified layers that have higher approximate symmetry than does the whole crystal; Baggio (2019 ▸, 2020 ▸) outlined a general method that can be used to find approximate symmetry of all types and applied it to a large number of *Z*′ = 4 structures.

l-DOPA is a major drug that has been used for more than 50 years to treat the motor symptoms of Parkinson’s disease; global sales of its various forms are measured in the billions of USD. It is then very surprising to find that the Cambridge Structural Database (CSD; Groom *et al.*, 2016 ▸) contains only two structures (FETTON and FETVEF; Suzuki *et al.*, 1998 ▸) in which l-DOPA is coordinated to a metal, and two more (XOYXUH and XOYXUH01; Shemchuk *et al.*, 2019 ▸) in which it is part of an ionic cocrystal (with LiCl).

There are numerous structures in the CSD of metal complexes containing the naturally occurring amino acids tyrosine (Tyr; 4-hy­droxy-l-phenyl­alanine) and phenyl­alanine (Phe) that are the biological precursors of l-DOPA. In almost all of those structures the amino acid coordinates through both its amino and carboxylate groups. The CSD version of May 2021 includes 61 different *R* ≤ 0.075 structures of metal complexes of Tyr and 83 of Phe; there are, however, only two structures of any precision of l-DOPA complexes. Given its medical significance and market value it seems certain that many attempts have been made to grow diffraction-quality crystals of metal complexes of l-DOPA, but if so, then most of those attempts failed. It would seem that the addition of the second hy­droxy substituent on the phenyl ring must be determining, but why?

O’Brien *et al.* note that they had difficulty finding a crystal that diffracted well. Shemchuk *et al.* (2019 ▸) mention the low quality of the data for XOYXUH; their structure of the polymorph XOYXUH01 was determined from powder data. The *R* factors for FETTON and FETVEF (0.073 and 0.079, respectively; Suzuki *et al.*, 1998 ▸) are surprisingly high. The small number of l-DOPA structures with metals and the problems with data quality suggest that some feature of l-DOPA inter­feres with crystal packing. Looking at the hydrogen-bonding tendencies of *vicinal* hy­droxy groups located on phenyl rings might be a way to approach the problem.

The reported structure is particularly unusual in having two amino acid ligands. Of the 61 Tyr complexes in the CSD, only 18 have two Tyr ligands; of the 83 Phe complexes, only 9 have two Phe ligands. In many of those structures, especially those containing Cu^2+^, the free O atom of a carboxyl­ate group from a neighboring mol­ecule completes the fivefold coordination sphere consistent with a *d* ^9^ ion capable of exhibiting a Jahn–Teller distortion. Sometimes, two such O atoms complete a sixfold coordination sphere. In the structure reported by O’Brien *et al.*, it is the 3-hy­droxy O atom on the phenyl ring that fills this role, although one of the independent Cu—O distances is considerably shorter than the other (2.74 *versus* 2.97 Å). In none of the 18 *M^n^* ^+^(Tyr)_2_ *L* complexes is the phenyl-ring OH group coordinated in that way.

The other notable feature of this structure is its approximate symmetry. There are hydrogen-bonded columns of mol­ecules along *c* that have easily recognizable, although quite distorted, 4_2_ axes. O’Brien *et al.* explain how to find and qu­antify this relationship using the *MATCH* routine in *Crystals for Windows* (Betteridge *et al.*, 2003 ▸) along with software they developed locally. The *FIT* routine in *PLATON* (Spek, 2020 ▸) gives essentially the same results for the rotation angle, the rotation axis, and the r.m.s. deviation (rmsd) for the best fit of the two mol­ecules. When using *PLATON* (and perhaps when using *MATCH*) it was necessary to remove the 3-OH substituents on the phenyl rings so that the program recognized the two complexes as independent rather than bonded, with each of them having approximate twofold symmetry.

Approximate symmetry relating independent mol­ecules is often associated with approximate symmetry that is periodic in at least two dimensions (Brock, 2020 ▸), but O’Brien *et al.* point out that the approximate 4_2_ axis does not lead to any supergroup description. In a three-dimensional supergroup inclu­ding a 4_2_ axis the crystallographic 2_1_ axis along *b* would have to be accompanied by an at least approximate 2_1_ axis along *a*, but along *a* there is no symmetry of any kind other than translation (Fig. 1 ▸). Layers having approximate symmetry that is periodic in two dimensions are also impossible because layer groups cannot include *n*-fold rotation or screw axes, *n* > 2, that lie within the layer or any screw axis perpendicular to the layer. Any such axis within the layer could relate mol­ecules through which it passes, but the axis would have to be local because if periodic it would move adjacent unit cells out of the layer.

The article concludes with a tribute to the first author, Professor Paul O’Brien of the University of Manchester, who passed away in 2018 while the manuscript was being formulated but before it could be completed. Since the delay was probably the result of the difficulty in finding a crystal that gave an acceptable diffraction pattern, the authors are to be applauded for their persistence. The finished article is a fitting tribute to Paul O’Brien.

## Figures and Tables

**Figure 1:**
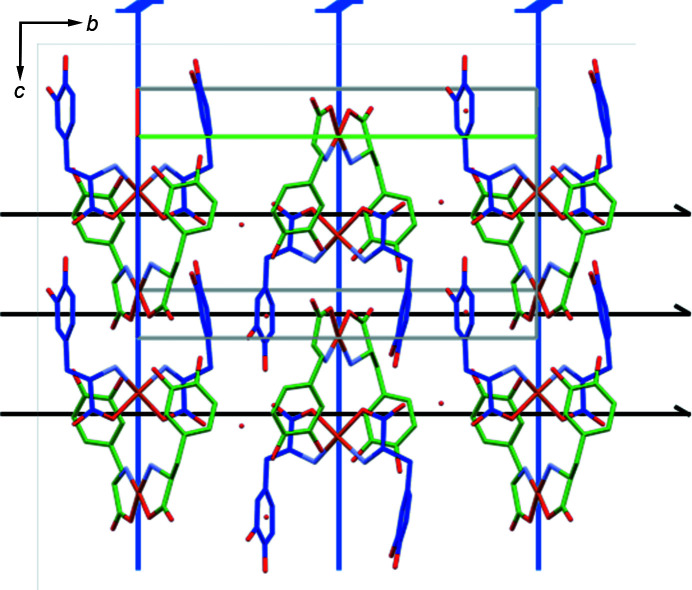
Projection along a* of part of a layer of molecules having centroids in the range 0 < *x* < 1. The projection of the *a* axis is visible because β = 104.7°. The crystallographic 2_1_ axes are shown in black and the approximate 4_2_ axes are shown in blue.
